# Cross-Cultural Revision and Psychometric Properties of the Chinese Version of the Autism Spectrum Rating Scale (2–5 Years)

**DOI:** 10.3389/fneur.2018.00460

**Published:** 2018-06-27

**Authors:** Hao Zhou, Chunpei Li, Xuerong Luo, Lijie Wu, Yi Huang, Lan Zhang, Xiaobing Zou, Xiu Xu, Yong-Hui Jiang, Weili Yan, Yi Wang

**Affiliations:** ^1^Department of Neurology, Children's Hospital of Fudan University, Shanghai, China; ^2^Guizhou Provincial People's Hospital, Medical College of Guizhou University, Guiyang, China; ^3^Department of Psychiatry, The Second Xiangya Hospital of Central South University, Changsha, China; ^4^School of Public Health, Harbin Medical University, Harbin, China; ^5^Department of Psychiatry, West China Hospital of Sichuan University, Chengdu, China; ^6^Department of Child Health, Chengdu Women and Children's Hospital, Chengdu, China; ^7^Child Development Center, The Third Affiliated Hospital, Sun Yat-Sen University, Guangzhou, China; ^8^Department of Child Health, Children's Hospital of Fudan University, Shanghai, China; ^9^Division of Medical Genetics, Department of Pediatrics and Neurobiology, Duke University School of Medicine, Durham, NC, United States; ^10^Department of Clinical Epidemiology, Children's Hospital of Fudan University, Shanghai, China

**Keywords:** ASD, ASRS, early screening, cross culture, psychometric properties

## Abstract

**Background:** No sufficient biomarkers are available for early identification of autism in the general population. Currently, the diagnosis of ASD depends on behavioral assessments. A useful screening tool can help to detect early autistic symptoms and provide children an early opportunity for ASD-related interventions. This research aimed to assess cross-cultural adaptation and psychometric properties of the autism spectrum rating scale (ASRS) under the Chinese cultural environment.

**Methods:** Participants were recruited from 17 kindergartens and 5 special education schools across five cities (Shanghai, Guangzhou, Changsha, Chengdu, and Harbin) in China. A total of 2,181 kindergarten children and 207 ASD cases participated in this study. Mplus 7.03 was utilized to conduct exploratory factor analysis, followed by adaptive modifications to construct the revised Chinese version of the ASRS (RC_ASRS).

**Results:** The result showed that 62 items comprised a two-factor structure; Factor 1 (social communication, SC) included 21 items, and Factor 2 (unusual behavior, UB) included 41 items. Cronbach's alpha ranged from 0.87 to 0.91 within the RC_ASRS. The total score and the SC and UB scores were significantly higher in ASD cases than in kindergarten samples (Cohen's d ranged from 0.82 to 2.72). The total RC_ASRS score showed an area under the curve (AUC) of 0.95 (95% CI: 0.93–0.97). With a total score cut-off ≥ 60, the RC_ASRS is an excellent tool to identify ASD cases from Chinese kindergarten children (sensitivity = 88.6%, specificity = 84.5%).

**Conclusions:** The RC_ASRS has excellent psychometric properties and is a reliable, useful tool for early ASD screening among Chinese children.

## Introduction

Autism spectrum disorder (ASD) is a cluster of neurodevelopmental disorders that develop in early childhood and are characterized by impaired social interactions and repeated stereotypic behaviors (1). ASD is a public health problem worldwide due to its significantly increased prevalence. Furthermore, ASD severely impacts the quality of life and places a substantial economic burden on individuals, families and society (2, 3). Currently, behavioral intervention is the primary treatment (4). Early intervention improves the prognosis of ASD (5–7). However, most children with ASD receive their first diagnosis when they enter the diverse school environment, which might be later than the optimal intervention age. Therefore, early identification and intervention are urgently needed for this population (8).

Currently, the diagnosis of ASD depends on behavioral assessment, because sufficient biomarkers are not available for the early identification of autism conditions in the general population. Experts have developed scales by combining both qualitative and quantitative methods based on the core symptoms of autism to help improve identification of ASD. These scales include the Checklist for Autism in Toddlers-23 (CHAT-23), the Social Responsiveness Scale (SRS), the Social Communication Questionnaire (SCQ), the Autism Behavior Checklist (ABC), the Childhood Autism Spectrum Test (CAST), and the Autism Spectrum Rating Scale (ASRS) (9–14). Although many tools are available, current ASD screening primarily focuses on children older than 5 years of age, whereas tools targeting children 2–5 years of age are lacking.

The ASRS was developed by Dr. Goldstein and Naglieri in 2009 and is available in two versions for young children 2–5 and 6–18 years of age (https://www.mhs.com). The ASRS shows excellent reliability and validity for ASD evaluation in the English-speaking population in the U.S. In our previous study (14), a systematic analysis of the Chinese version of the ASRS among 6-to-18-year-old children was conducted using community-based ASD cases. Notably, the appropriate revised Chinese version of the ASRS (6–18 years) had excellent psychometric properties and achieved a sensitivity of 94.2% and a specificity of 82.0% for ASD screening in Chinese children (15). Based on our previous results, this study focused on introducing the ASRS early screening version (2–5 years) and used exploratory factor analysis to evaluate its psychometric properties in the Chinese cultural background.

## Methods

### Samples

The samples included two subsets from five cities collected from January 2016 to October 2016.

Children were recruited from 5 cites in China (Shanghai, Guangzhou, Changsha, Chengdu, and Harbin) from the enrolled members of a national epidemiological study of ASD in China, which was supported by the National Health and Family Planning Commission of the People's Republic of China (201302002). A total of 2,181 children from the general population aged 2–5 years were enrolled in this study from 17 kindergartens, and 207 clinically diagnosed ASD cases aged 2–5 years were recruited from special education schools across the five study sites to analyze the reliability and validity of the Chinese version of the ASRS. All recruited ASD cases met the DSM-5 diagnostic criteria, and the clinical diagnoses of ASD were confirmed by a pediatric psychiatrist at the research institutions (Children's Hospital of Fudan University, The Third Affiliated Hospital of Sun Yat-Sen University, West China Hospital of Sichuan University, Chengdu Women and Children's Hospital, The Second Xiangya Hospital of central South University, and the Harbin Medical University), which are authorized ASD diagnostic centers in China. Caregivers of all recruited children and ASD cases were invited to complete the Chinese version of the ASRS following a standard protocol.

### Chinese version of the ASRS

Our team adopted standard translation and back-translation procedures to develop the Chinese version of the ASRS with permission from the Multi-Health System (https://www.mhs.com). We recruited a few parents of ASD cases aged 2–5 years from the outpatient clinic of Children's Hospital of Fudan University to complete the Chinese version of the ASRS. All participants believed that the context of the Chinese version of the ASRS was understandable.

The 70-item pool of the ASRS was established based on the core symptoms of autism. Each item's response was measured using a 5-point Likert scale (“0” indicating never and “4” indicating very frequently) to quantify autistic symptoms. The ASRS includes three scales (the ASRS, DSM-5, and treatment scales) with different items forming the 70-item pool based on the specific purpose. The ASRS scales include two subscales comprising 62 of the 70 total items used for screening: Social/Communication (39 items, SC) and Unusual Behaviors (23 items, UB). The two subscales were combined into a single composite score (the total score), which was used for ASD screening among U.S. children. This study mainly focused on the ASRS scales with an exploratory factor analysis.

The DSM-5 scales consist of 35 of the 70 items according to the consensus ASD expert group and play an auxiliary role in the diagnosis of ASD. The treatment scales include the following 8 subscales: Peer Socialization (9 items, PS), Adult Socialization (5 items, AS), Social/Emotional Reciprocity (12items, SER), Atypical Language (6 items, AL), Stereotypy (6 items, ST), Behavioral Rigidity (8 items, BR), Sensory Sensitivity (6 items, SS), and Attention/Self-Regulation (10 items, ASR). The items in each subscale are selected from the 70-item pool by the consensus ASD expert group. These scales can be used to monitor the behavior intervention response for children with ASD.

The raw scores of each scale (ASRS, DSM-5, and treatment scales) were converted to standardized scores to facilitate interpretation of the results and comparisons with previous studies.

### Procedure

The caregivers were invited to provide consent and complete the Chinese version of the ASRS under guidance of screening booklets. Contact information, including the telephone numbers and e-mail addresses of the research team, were provided to help with questionnaire collection. This study was approved by the Children's Hospital of Fudan University Ethics Board ([2012] No. 185).

### Statistical analyses

The statistical package Mplus 7.03 (Muthén & Muthén, Los Angeles, CA, USA) was employed to perform the data analysis. An exploratory factor analysis (EFA) was used to examine the latent model structures. The model estimation was completed using robust weighted least squares means and variance adjustment (WLSMV) (16). The factor structure was estimated with the Chi-square goodness-of-fit test, root mean square error of approximation (RMSEA), comparative fit index (CFI), Tucker-Lewis index (TLI), and standardized root mean square residual (SMSR)(17). The number of factors retained in the model was determined by the scree test (18). Factors with loadings >0.3 or differences in cross-loadings >0.1 were retained, whereas all other items were removed from the model.

Item reliability was analyzed using Cronbach's alpha. We compared the mean scores of the ASRS between the ASD cases and the kindergarten children by using Student's *t*-test to measure the discriminant validity and calculated Cohen's *d* value to test between-group differences (effect size). The area under the curve (AUC) and 95% confidence intervals (95% CIs) were calculated to evaluate the overall performance of the questionnaire. The sensitivity and specificity values for the discrimination of children with ASD from the general population in the study samples were assessed based on the recommended cut-off total score of 60. All tests were two-tailed, and a *P-*value of 0.05 was retained as the level of statistical significance.

## Results

### Sample demographic characteristics

In total, 2,181 ASRS questionnaires from 17 kindergartens and 207 from 5 special education school were collected. A total of 430 (19.7%) questionnaires from kindergartens and 40 (19.3%) questionnaires for ASD cases were not included in the final analysis due to a missing item or basic information (e.g., name and date of birth). Finally, a total of 1,751 questionnaires from the kindergartens and 167 from the ASD cases were included in the factor analysis. The mean age of the kindergarten children was 4.0 ± 0.8 years, and the male to female ratio was close to 1.08:1. In contrast, the mean age of the ASD children was 3.3 ± 1.1 years, and the male to female ratio was 6.95 to 1 (Table 1). The mean ages and sex ratios were significantly different between the two groups (*P* < 0.001).

**Table 1:** Basic information for the samples.

**Characteristics**	**Category**	**Kindergarteners (*n* = 1,751)**	**ASD cases (*n* = 167)**	***P*-value**
		**Mean (SD) or *n*(%)**	**Mean (SD) or *n*(%)**	
Age	Mean	4.0 (0.8)	3.3 (1.1)	<*0.001*
Gender	Male	910 (52.0)	146 (87.4)	<*0.001*
	Female	841 (48.0)	21 (12.6)	
Rater	Father	505 (28.8)	43 (25.7)	0.636
	Mother	755 (43.1)	71 (42.5)	
	Others	20 (1.1)	3 (1.8)	
	Missing	471 (27.0)	50 (30.0)	
Rater's education	Middle school	125 (7.1)	34 (20.4)	<*0.001*
	Vocational	669 (38.2)	67 (40.1)	
	Bachelor's	656 (37.5)	52 (31.1)	
	Master's	283 (16.2)	12 (7.2)	
	Missing	18 (1.0)	2 (1.2)	
Father's occupation	Farmer	291 (16.6)	40 (24.0)	0.135
	Worker	448 (25.6)	46 (27.5)	
	Manager	217 (12.4)	16 (9.6)	
	Technician	314 (17.9)	24 (14.4)	
	Other	451 (25.8)	41 (24.5)	
	Missing	30 (1.7)	0 (0.0)	
Mother's occupation	Farmer	252 (14.4)	37 (22.2)	0.002
	Worker	501 (28.6)	34 (20.4)	
	Manager	171 (9.8)	9 (5.4)	
	Technician	187 (10.7)	13 (7.8)	
	Other	609 (34.8)	70 (41.9)	
	Missing	31 (1.7)	4 (2.3)	

*Italic values mean that the difference between the two groups was significant*.

### Exploratory factor analysis

**Figure 1:**
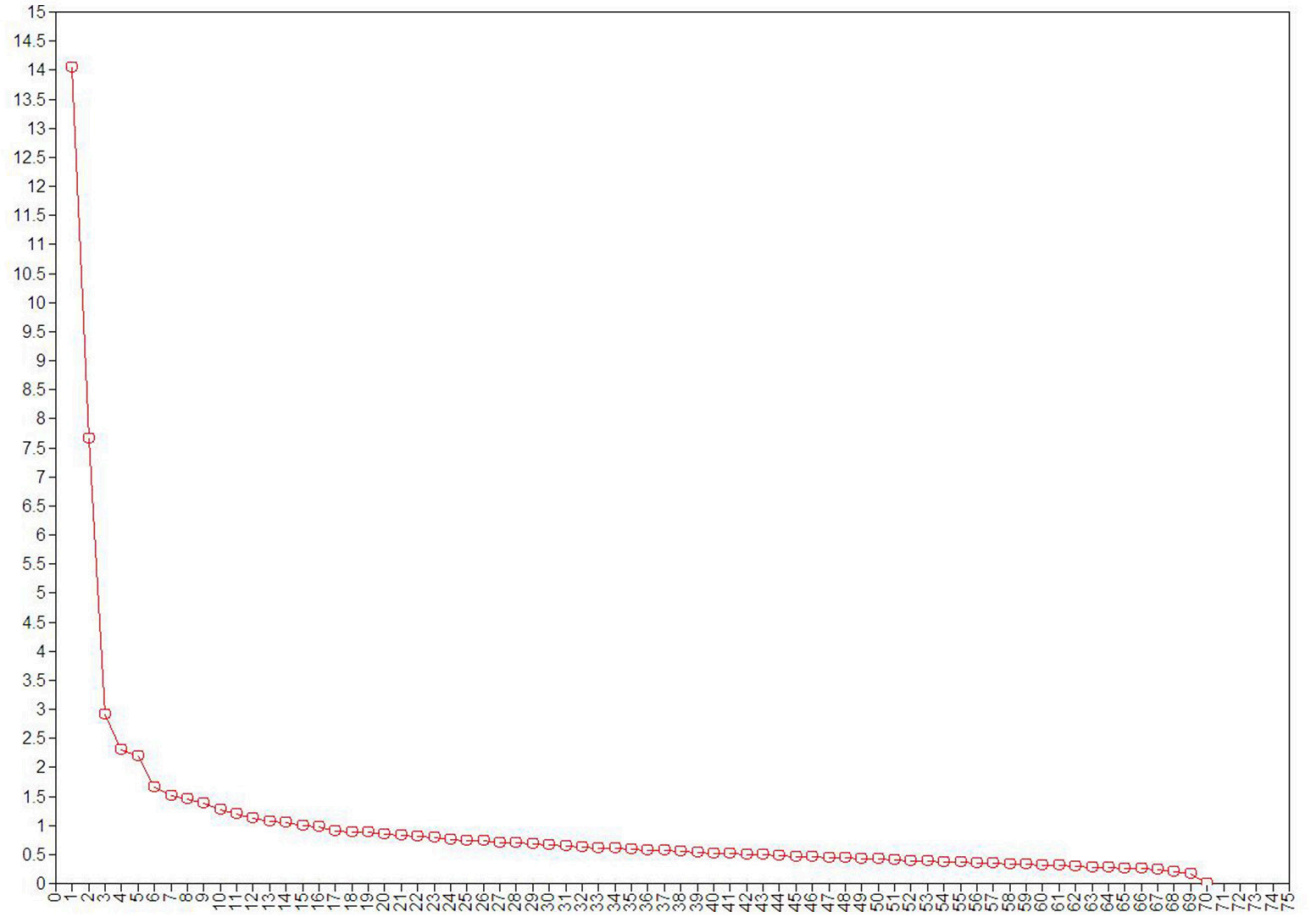
Scree plot. We selected the number of factors for retention via the scree test where components with eigenvalues before the “break” of a scree plot were retained.

The scree test was performed to determine the number of factors for inclusion in the Chinese version of the ASRS. Although a break was apparent in the slopes of the plotted eigenvalues, the shape of the curve suggested that 2 factors were appropriate for the current samples, as shown in Figure 1. According to the core domain of ASD, the chosen two-factor structure may be suitable for the Chinese version of the ASRS with the following model fit factors: RMSEA = 0.059, CFI = 0.81, TLI = 0.80, and SRMR = 0.06.

Eight of the 70 items were excluded due to factor loadings <0.3 or cross-loading differences <0.1; the loadings of all items are shown in Table S1. The 62 items comprised two factors, and the total numbers and factor names were similar to those of the unrevised Chinese version of the ASRS (C_ASRS). However, the number and context of each factor in the revised Chinese version of the ASRS (RC_ASRS) significantly differed from those of the C_ASRS (Factor 1: 21 vs. 39 items, Factor 2: 41 vs. 23). Factor 1 (social communication, SC) now included 21 items (1, 3, 4, 5, 13, 14, 16, 19, 21, 25, 28, 29, 35, 38, 40, 50, 52, 54, 56, 57, and 61), whereas factor 2 (unusual behavior, UB) included 41 items (2, 6, 9, 10, 12, 15, 17, 20, 22, 23, 24, 26, 27, 30, 31, 32, 33, 34, 37, 41, 42, 43, 44, 45, 46, 47, 48, 51, 53, 55, 58, 59, 60, 62, 63, 64, 65, 66, 67, 68, and 69).

### The RC_ASRS scores in kindergarten children

The total scores were 50.27 ± 10.59 vs. 50.45 ± 10.19 based on the fathers' and mothers' ratings, respectively. No significant differences (*P* = 0.785) were observed. The average total scores were 50.72 ± 10.02 vs. 49.56 ± 10.26 for boys and girls, respectively. Boys had slightly higher scores on all subscales (Table 2). All subscale scores had slight differences across sites (All *P* < 0.001, see Table S2).

**Table 2:** Gender differences in the RC_ASRS scores of kindergarteners.

**ASRS scale**	**Boys (*n* = 910)**	**Girls (*n* = 841)**	***t-*value**	***P-*value**
SC	50.62 ± 10.14	49.44 ± 10.00	2.439	0.015
UB	50.57 ± 10.09	49.85 ± 10.65	1.444	0.149
Total score	50.72 ± 10.02	49.56 ± 10.26	2.396	0.017

*SC, Social/Communication; UB, Unusual behaviors*.

### Item reliability

Cronbach's alpha was used to measure the item reliability for the RC_ASRS (19); the values were 0.91 for all 62 items of the RC-ASRS, 0.87 for SC, and 0.91 for UB. The item reliability results revealed that the item structure of the RC_ASRS was robust and reasonable. The RC_ASRS was associated with a slightly higher Cronbach's alpha than the C_ASRS among Chinese kindergarten children, especially for the UB and total scores, as shown in Table 3.

**Table 3:** Analysis of item reliability for the RC_ASRS and C_ASRS.

**Factors**	**RC_ASRS**	**Cronbach's alpha**	**C_ASRS**	**Cronbach's alpha**
SC	21	0.87	39	0.89
UB	41	0.91	23	0.80
Total score	62	0.91	62	0.89

*RC_ASRS, revised Chinese version of the ASRS; C_ASRS, unrevised Chinese version of the ASRS; SC, Social/Communication; UB, Unusual behaviors*.

### Discriminant validity

To test the discriminant ability of the RC_ASRS and C_ASRS, we compared the mean scores between kindergarten children and ASD cases (Table 4). The total, SC, and UB scores of the RC_ASRS were significantly higher for the ASD cases than for the kindergarten children (Cohen's d ranged from 0.82 to 2.72). In contrast, the UB scores of the C_ASRS were significantly higher for the kindergarten children than for the ASD cases (the Cohen's *d*-value was <0.00).

**Table 4:** Discriminant validity of the RC_ ASRS.

	**ASRS scale**	**Kindergarteners (*n* = 1,751)**	**ASD cases (*n* = 167)**	***t***	***P-*value**	**Cohen's *d***
RC_ ASRS	SC	50.05 ± 10.09	80.79 ± 12.34	−*31.21*	<*0.001*	2.72
	UB	50.23 ± 10.37	58.59 ± 10.13	−*9.99*	<*0.001*	0.82
	Total score	50.17 ± 10.15	74.20 ± 11.01	−*29.00*	<*0.001*	2.27
C_ ASRS	SC	50.06 ± 10.12	83.27 ± 11.95	−*34.74*	<*0.001*	3.00
	UB	50.00 ± 10.51	41.34 ± 12.56	*8.625*	<*0.001*	−0.75
	Total score	50.03 ± 10.35	65.30 ± 11.35	−*18.05*	<*0.001*	1.41

*RC_ASRS, revised Chinese version of the ASRS; C_ASRS, unrevised Chinese version of the ASRS; SC, Social/Communication; UB, Unusual behaviors. Italic values mean that the difference between the two groups was significant*.

### ROC analysis

**Figure 2:**
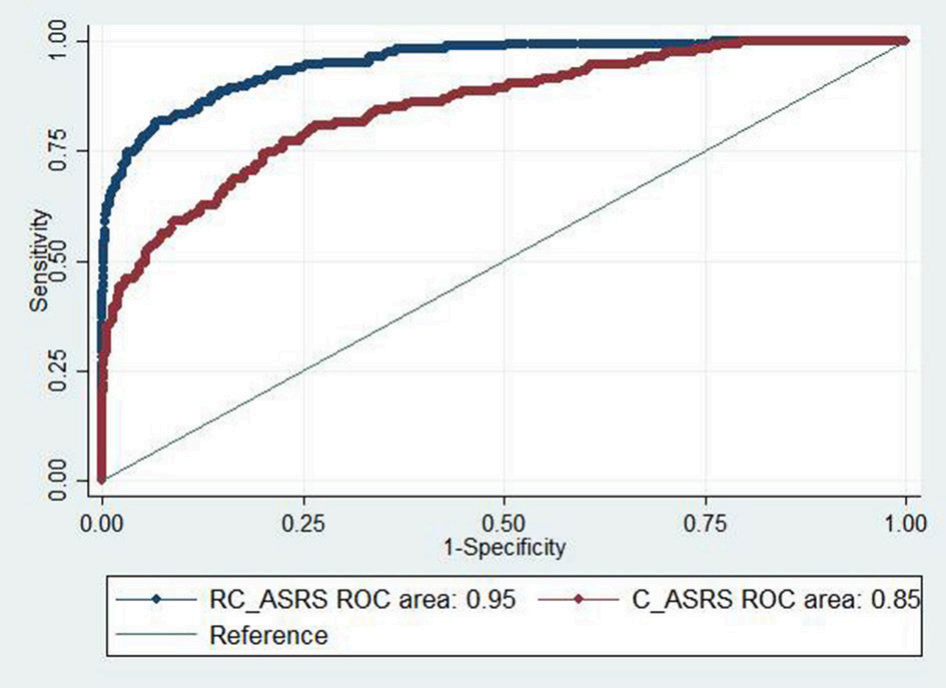
Receiver operating characteristic (ROC) curves for the total scores for the RC_ASRS and C_ASRS.

The RC_ASRS had a total score AUC of 0.95 (95% CI: 0.93–0.97) vs. 0.85 (95% CI: 0.82–0.88) for the C_ASRS. The results indicated that the discriminant validity of the RC_ARSR for ASD screening in kindergarten children was significantly higher than that of the C_ASRS (Figure 2). The same analysis conducted by comparing differences among sexes showed equal performance among the boys and girls (AUC = 0.95; 95% CI: 0.93–0.97 vs. AUC = 0.94; 95% CI: 0.90–0.99) for the ASRS. A higher AUC was obtained for the mother raters than for the father raters (AUC = 0.97; 95% CI: 0.96–0.99 vs. AUC = 0.95; 95% CI: 0.92–0.98), although the differences was not significant.

A total score cut-off ≥60 for the RC_ASRS achieved the maximum Youden index (sensitivity, 88.6%; specificity, 84.5%). However, the same cut-off for the C_ASRS achieved comparable specificity but a poorer performance in sensitivity (sensitivity, 65.9%; specificity, 84.8%). The screening ability of the ASRS is slightly better among U.S. children than among Chinese children with the same cut-off (sensitivity, 88.6 vs. 89.8%; specificity, 84.5 vs. 90.3%) (https://www.mhs.com).

## Discussion

Autism assessment instruments are widely used for basic and clinical research on ASD. Most ASD scales developed to date are based on data obtained from the children of English-speaking populations, whereas few scales are based on data obtained from Chinese-speaking children. A previous study revealed that different cultural contexts could impact the performance of the scales (10). The ASRS is a relatively new ASD assessment tool with excellent reliability and validity in English-speaking children in the U.S. (https://www.mhs.com). In our previous study, we reported that the psychometric properties of the revised Chinese version of the ASRS (6–18 years) were better than the directly translated version tested in Chinese children. However, analysis of the factor structure and psychometric properties of the early version of the ASRS (2–5 years) remained unexplored. Our study addressed this problem and suggested that the RC_ASRS had excellent psychometric properties and was reliable and useful for ASD screening among Chinese children.

Factor analysis is a well-established method to determine the latent structures of questionnaires (20). This analysis was used to investigate the factor structure of the Chinese version of the ASRS in this study. To our knowledge, ASD symptoms are involved in two domains according to the DSM-5 manual. Ultimately, based on the shape of the scree plot, two factors were appropriate for the current sample. All model fit values were <0.9, which fell short of the optimal value in this study. Generally, the optimal value of the model was determined based on a theoretical model. The model fit rarely meets certain criteria, especially those composed of categorical variables (21). Thus, the inadequate model fit reached using the standard judgment criteria may have been due to the inclusion of categorical variables and the nonnormal distribution of the current data.

The factor name and the number of total items in the RC_ASRS were based on the EFA results, which were similar to the factor name and total items of the C_ASRS. One difference was the change in the item number and content of each factor. Several aspects might explain these changes; for example, items moved from the SC factor to the UB factor, and some items were added to the RC_ASRS from the item pool compared with the C_ASRS. Items should be removed or added with caution because unreasonable changes may affect the performance of a questionnaire. However, this difference may be reasonable, because diverse cultural aspects may have affected participants' understanding of some items. Some items belong to SC in the C_ASRS, such as item 15 (“Have trouble talking to other children”), item 17 (“Appear disorganized”), and item 22 (“Uses language that is immature for his or her age”). In contrast, these items were added to UB of the RC_ASRS. Previous reports have demonstrated the necessity of modifying questionnaires based on different cultures or backgrounds (22, 23).

To test the psychometric properties of the RC_ASRS, we conducted item reliability and discriminant validity analyses. The item reliability was slightly better for the RC_ASRS than for the C_ASRS. The item reliability data indicated that the factor components were robust. In this study, the C_ASRS screening subscales (e.g., UB) showed that the score was significantly higher among the general child population than among children with ASD, which demonstrated that the C_ASRS had poor discriminative validity. However, all subscale scores of the RC_ASRS were significantly higher among the children with ASD than among the kindergarten children, which demonstrated that the RC_ASRS had an excellent identification ability for autistic symptoms among children from the general population compared with the C_ASRS. As indicated by the high AUC values, the RC_ASRS showed a better ASD screening performance. Using the same cut-off, the RC_ASRS had much higher sensitivity (88.6 vs. 65.9%) and equal specificity values (84.5 vs. 84.8%) than did the C_ASRS. All of these data showed that the RC_ASRS was more suitable for ASD screening among Chinese children than the C_ASRS.

### Limitations

The samples were recruited across five cities in this study. Differences in language and economic levels exist in the current sample. The specific effect of each variable was not tested. The results should be interpreted with caution. First, EFA is a preliminary test, and the results must be confirmed with other samples. Second, due to missing data, all of the collected questionnaires were not included in the final analysis, but the study sample was sufficiently large, and the vast majority (80.0%) of questionnaires were included; thus, the deletion of missing data is unlikely to have affected the EFA results. The criteria for model fit and factor loading may also have affected the factor structure.

## Conclusions

In conclusion, 62 items comprised the two-factor structure of the RC_ASRS, which showed excellent item reliability and discriminate validity with higher sensitivity and specificity. The RC_ASRS has suitable psychometric properties; therefore, it is useful for early screening for autism among Chinese children.

## Author contributions

HZ and CL wrote the manuscript. HZ, CL, XL, LW, YH, LZ, and XZ collected the data. HZ and WY completed the data analysis. WY, Y-HJ and YW revised the manuscript. XL, LW, YH, LZ, XZ, XX, WY, and YW conducted and designed the study.

### Conflict of interest statement

The authors declare that the research was conducted in the absence of any commercial or financial relationships that could be construed as a potential conflict of interest. The handling Editor declared a shared affiliation, though no other collaboration, with several of the authors (HZ, CL, XX, WY, and YW).
