# Supplementary material for: Cross-Cultural Revision and Psychometric Properties of the Chinese Version of the Autism Spectrum Rating Scale (2–5 Years)
Source: Front Neurol. 2018 Jun 27;9:460. doi: 10.3389/fneur.2018.00460 (PMC6036302; doi:10.3389/fneur.2018.00460)
Supplement: Supplementary file 1 [file Table_1.PDF]

Table S1 The factor loadings of item for Chinese version of the ASRS

Item	Factor 1	Factor 2
1.smile appropriately?	0.566*	-0.040
2.become bothered by some fabrics or tags in clothes	-0.089	0.329*
3.understand how someone else felt?	0.616*	-0.027
4.play with others?	0.634*	-0.056
5.look at others when talking with them?	0.628*	0.021
6.ask questions that were off topic?	-0.230	0.449*
7.point to objects when asked to?	-0.465	-0.305
8.insist on doing things the same way each time?	0.326*	0.379*
9.need things to happen just as expected?	-0.254	0.486*
10.have a strong reaction to any change in routine?	-0.164	0.615*
11.line up objects in a row?	0.364*	0.337*
12.overract to common smells?	-0.100	0.480*
13.look at others when interacting with them?	0.696*	-0.052
14.understand the point of view of others?	0.641*	0.004
15.have trouble talking with other children?	-0.425	0.420*
16.share fun activities with others?	0.596*	0.006
17.appear disorganized?	0.185	0.428*
18.use make believe play?	-0.381	-0.317
19.care about what other people think or feel?	0.638*	-0.083
20.become upset if routines were changed?	-0.078	0.566*

21.respond when spoken to by adults?	0.561*	0.059
22.use language that was immature for his/her age?	-0.084	0.481*
23.avoid looking at an adult when there was problem?	0.004	0.451*
24.choose to play alone?	-0.299	0.352*
25.listen when spoken to?	0.581*	0.021
26.talk too much about things that other children don’t care about?	-0.061	0.546*
27.focus too much details?	-0.195	0.527*
28.start conversations with others?	0.603*	0.021
29.keep a conversation going?	0.676*	0.004
30.play next to, but not with, other children?	-0.366	0.375*
31.get into trouble with adults?	-0.426	0.492*
32.fail to complete tasks?	0.272	0.433*
33.have special problems with adults?	-0.484	0.484*
34.have problems waiting his/her turn?	0.304	0.422*
35.play with toys appropriately?	0.444*	0.119
36.show little emotion?	-0.364	0.241
37.learn simple tasks but then forget them quickly?	0.222	0.342*
38.notice social cues?	0.409*	-0.189
39.become fascinated with parts of objects?	0.019	-0.490
40.respond when spoken to by other children?	0.632*	0.013
41.talk too much about things that adults don’t care about?	-0.180	0.502*

42.use an odd way of speaking?	0.021	0.611*
43.avoid looking at people who spoke to him/her?	0.312	0.508*
44.have trouble talking with adults?	-0.448	0.517*
45.resist being touched or held?	0.235	0.405*
46.overreact to loud noises?	-0.005	0.542*
47.focus on one subject for too much time?	0.046	0.527*
48.insist on keeping certain objects with him/her at all times?	0.024	0.454*
49.seek the company of other children?	0.302*	0.266
50.ahow an interest in the ideas of others?	0.529*	-0.150
51.have social problems with children of the same age?	-0.331	0.397*
52.undrstand age-appropriate humor or jokes?	0.563*	0.008
53.repeat certain words or phrases out of context?	0.004	0.558*
54.share his/her enjoyment with others?	0.676*	-0.012
55.have problems playing attention to fun tasks?	0.304*	0.434*
56.insist on certain routines?	0.409*	0.027
57.follow instructions that he/she understood?	0.560*	0.069
58.inerrupt or intrude on others?	-0.065	0.383*
59.reverse pronouns(e.g., you and me)?	0.226	0.389*
60.become obsessed with details?	-0.073	0.515*
61.show good peer interactions?	0.600*	0.080
62.appeat fidgety when asked to sit still?	0.186	0.513*
63.become distracted?	0.145	0.447*

64.flap his/her hands when excited?	-0.288	0.388*
65.twirl, spin, or bang objects?	-0.231	0.481*
66.smell,taste or eat inedible objects?	0.006	0.515*
67.fail to make his/her needs known?	0.324	0.436*
68.hurt him/herself(e.g., banged own head) when upset?	0.178	0.419*
69.overract to touch?	0.204	0.499*
70.repeat or echo what others said?	-0.465	-0.305

Note: * factor loadings >0.3, an item was removed if it had a factor loading<0.3 or cross-loading<0.1. Among the 70 items, 8 were excluded: items 7 (point to objects when asked to?), item 8 (insist on doing things the same way each time?), item 11 (line up objects in a row?), item 18 (use make believe play?), item 36 (show little emotion?), item 39 (become fascinated with parts of objects?), item 49 (seek the company of other children?), item 70 (repeat or echo what others said?)

Table S2 Site differences in the RC_ASRS scores among kindergarteners

ASRS scale	Shanghai (n=173)	Harbin (n=390)	Guangzhou (n=166)	Changsha (n=337)	Chengdu (n=685)	*F*	*P*-value
SC	47.32±9.85	49.33±10.03	47.39±8.59	50.03±9.64	51.81±10.41	*12.07*	*<0.001*
UB	48.62±11.50	49.52±11.46	47.87±10.01	51.68±9.69	50.89±9.64	*6.6076*	*<0.001*
Total score	47.50±10.78	49.28±10.67	47.08±9.68	51.05±9.27	51.65±9.89	*12.182*	*<0.001*

Social/Communication (SC), Unusual behaviors (UB)
